# A Randomized Controlled Trial Assessing the Psychological Benefits of a Daily Examen-Based Practice

**DOI:** 10.1007/s10943-025-02259-w

**Published:** 2025-01-25

**Authors:** Thomas G. Plante, David B. Feldman, Jacqueline Ge, Anthony Cortese

**Affiliations:** Department of Psychology, Santa Clara University, Santa Clara, CA 95053-0333 USA

**Keywords:** Meditation, Prayer, Gratitude, Spiritual practices, Catholic, Jesuit

## Abstract

This is a randomized controlled trial of an Examen-based practice, an intervention reflecting a five-step daily reflection and prayer practice developed by St. Ignatius of Loyola, founder of the Catholic Jesuit order. Like other practices (e.g., mindfulness, yoga), this practice can be used as a spiritual or secular intervention to help people with a variety of challenges and stressors. In this exploratory study, 57 university students were randomly assigned to a two-week daily Examen-based condition, while 58 students were assigned to a wait-list control condition. Questionnaires measuring hope, life meaning, satisfaction with life, mindfulness, compassion, stress, anxiety, and depression were administered pre- and post-intervention and subsequently at two-week follow-up. Significant differences were found for conditions on the measures of life meaning, satisfaction with life, and hope, suggesting that the Examen-based practice produces improvements in individuals’ global evaluations of their lives as well as their perceptions of the future. Suggestions for further research are offered.

## Introduction

The Examen is a brief structured prayer and reflection practice originating from the Roman Catholic tradition. Developed in the fifteenth century by St. Ignatius of Loyola (Manney, 2011; Martin, 2021; Mottola, 1964), founder of the Jesuit order (Barry & William, 2014; Martin, 2013; O'Malley, 1993), the Examen typically consists five steps requiring approximately 15 min to complete. It was designed to be practiced at the end of each day, with the individual reflecting upon the day just finished. The first step involves placing oneself in God’s presence, or in the presence of the “sacred,” however understood by the participant. In some secular versions of the Examen (i.e., Examen-based practices), this involves simply grounding oneself in the present moment. The second step consists of giving thanks or being grateful for the moments and experiences of one’s day. The third step involves reviewing the experiences of the day, reflecting on what went well and not-so-well, while remaining mindful that the “divine” or “sacred” is present in all these moments. The fourth step encourages a reflection on one’s shortcomings, with consideration given to how improvements can be made in the future. Finally, the practice is concluded by planning for the next day, reflecting on and learning from the review of the current day.

While the Examen is popular among Christians, it can easily be adapted to accommodate the needs and values of a religiously pluralistic audience, including secular individuals (see Table 1; Plante, 1999, 2008, 2017). In fact, the Examen and Examen-based practices have been used by diverse populations, including both religious and non-religious communities, and have been examined for their psychological benefits (Chinnici, 1997; Curlee & Ahrens, 2022; Frederick et al., 2021; Martin, 2013; Proeschold-Bell et al., 2023; Tetlow, 1994). The Examen and Examen-based interventions have also been utilized as tools in various types of mental health treatment, including for couples therapy (Priester, 2006), work burnout (Case et al., 2020; Frederick et al., 2021; McMillin, 2021), stress management (Proeschold-Bell et al., 2023), and substance use (Buenrostro & Plante, 2024).

**Table 1:** Five steps of the Examen, religious, and secular

Religious version	Secular version
1. Put self in God’s presence	1. Quiet and center the mind with silence
2. Give thanks to God	2. Be grateful
3. Review the day attending to God’s presence	3. Review the day attend to good moments
4. Attend to daily shortcomings	4. Attend to daily shortcomings
5. Invite God to be with you tomorrow	5. Plan for tomorrow and to do better

Spiritual practices—of which the Examen is one—are employed by many individuals for comfort, support, and healing (e.g., Pargament, 2007; Plante, 2009, 2024a, 2024b). These techniques may include prayer, ritual, meditative and contemplative practices, sacred music, liturgies, fasting, charitable almsgiving, and spiritual counseling, among numerous other activities. Many of these strategies have been secularized to meet the needs of a more general population.

The best example of the secularization of religious or spiritual practices is mindfulness meditation. Mindfulness has become popular in recent decades throughout the Western world (e.g., Germer et al., 2013; Khoury et al., 2013). While mindfulness emerged from the Buddhist tradition, it has been secularized to appeal to individuals of diverse spiritual traditions as well as those practicing no spiritual tradition. People often embrace and practice mindfulness without any direct connection to Buddhism (Barker, 2014; Plante, 2016).

Yoga offers another example of the secularization of a religious or spiritual practice. Yoga derives from the Hindu tradition. However, a version of yoga has become essentially an “exercise class,” offered in health clubs, spas, and studios for secular clients (Khalsa, 2013; Smith, 2011). Curiously, expensive “yoga wear” (e.g., Lululemon) has become as much a symbol of yoga as the actual yoga postures (Lozanski & Lavrence, 2019; Webb et al., 2017).

The secularization of spiritual or religious practices is not without its detractors, for good reason. Scholars have expressed concerns about cultural appropriation, where religious practices and symbols have been used in ways that were not intended by the originating traditions. Additionally, some worry that appropriation could be used for profit-making purposes (Bregman, 2019; Brunk & Young, 2009; Nardella, 2012; Plante, 2016). Thus, such secularization should always be undertaken with great care and respect (Brunk & Young, 2009; Buenrostro & Plante, 2024).

### The Present Study

In the present research, we conducted an exploratory randomized controlled trial on the use of a secularized version of the Examen—which we call an “Examen-based practice”—among a secular audience of university students. The purpose of this study was to undertake an initial exploration of the possible effects of an examine-based practice on a relatively wide array of outcomes. It involved randomly assigning students to either a two-week daily Examen-based practice condition or a wait-list control condition. We measured a variety of well-being-related outcomes immediately pre- and post-intervention, as well as at two-week follow-up.

We hypothesized that engaging in an Examen-based practice would result in several psychological benefits relative to those in the control condition. First, we measured hope, defined as a general, positive, goal-directed expectancy (Snyder, 1994; Snyder et al., 1991). In particular, the State Hope Scale (SHS; Snyder et al., 1996) assesses individuals’ perceptions of their abilities to develop plans (i.e., pathways) and conjure motivation (i.e., agency) to achieve life goals. Given that the Examen-based practice incorporates planning and considering one’s aspirations or goals for the following day, we expect that it will lead to higher hope.

Another relevant construct is mindfulness, often defined as present-moment awareness, attention, and acceptance (Brown & Ryan, 2003). With the Examen-based practice’s emphasis on active reflection, awareness of the spiritual or divine in the moment, and the experience of gratitude for recent events, we hypothesize that it will positively impact mindfulness.

We also measured both life meaning and life satisfaction. Meaning in life has been shown to relate to both satisfaction and religiousness (Steger & Frazier, 2005). The Examen-based practice, derived from religious or spiritual roots, encourages reflection on one’s experiences and a consideration of potential improvements that may lead to greater meaning. Relatedly, the practice’s emphasis on personal reflection, including its encouragement to consider aspects of the day that one is grateful for, may increase general satisfaction with life (Diener et al., 1985).

In addition, we measured compassion as a possible outcome of participating in the Examen-based practice. Compassion has been defined as an attitude of understanding and caring (Hwang et al., 2008). Such an attitude may also be addressed through the Examen-based practice’s reflection process, as participants are encouraged to ponder daily interactions and how they may have fallen short of their ideals.

Finally, while many of the aforementioned constructs are related to well-being, symptoms of depression, anxiety, and stress were also examined. Given that scholars have speculated that Examen-based practices may have therapeutic benefits (McMillin, 2021; Priester, 2006), it would be valuable to assess whether participating in the practice may decrease these prevalent mental health concerns.

## Method

### Participants

Participants included 115 students from a private, Jesuit-affiliated university in Northern California who took part in the study as one means of fulfilling the requirements of their introductory psychology courses. Participation was voluntary given that students also had non-research options for fulfilling these requirements, and participating students were informed that they could stop their participation at any time without consequence. There were no specific inclusion or exclusion criteria for beyond being at least 18 years of age. Sample characteristics can be found in Table 2. A total of 26 participants identified as male and 89 as female; no participants identified as gender non-binary or any other gender. Participants ranged in age from 18 to 22, with a mean of 18.85 (SD = 0.98). Sixty-eight students identified as White, 42 identified as Asian or Asian American, 18 identified as Latinx, 7 identified as Black or African American, and 3 identified as “other.” (Participants could indicate more than one racial or ethnic identity.) A power analysis shows that, though our sample was relatively small, it was sufficient to detect a medium effect, though not a small effect. To detect a medium effect (eta^2^ = 0.06; Cohen, 1988) with a power of 0.80, a total of 28 participants would be required. However, to detect a small effect (eta^2^ = 0.01), 162 participants would be needed.

**Table 2:** Participant demographic characteristics (*N* = 115)

	*Condition*		
	All	Examen	Control
*Gender*			
Male	26	11	15
Female	89	46	43
Non-binary	0	0	0
*Ethnicity*			
White	68	32	36
Asian	42	21	21
Latinx	18	9	9
Black	7	3	4
Other	3	3	0
*Age*			
Range	18–22	18–22	18–22
M (SD)	18.85 (.98)	18.81 (1.06)	18.90 (.89)

No statistically significant differences were found in participant demographics between experimental conditions

### Procedure

The research proposal and project were approved by the university’s Institutional Review Board (IRB) regarding ethical and legal compliance. Participants were randomly assigned to one of two conditions. Those in the experimental group (*n* = 57) received the Examen-based intervention, and those in the control group (*n* = 58) received no intervention for the duration of the study, but were invited to participate in the intervention once the study was completed.

All sessions were held online via Zoom. Students met for 15 min each evening at 8 pm, Sunday through Thursday, for a period of two weeks (i.e., 10 sessions in total), and were guided through the Examen-based practice (see the Appendix for the structured steps of the sessions). All sessions were led by the fourth author, an expert in the Examen and in Jesuit spirituality, more broadly. As an attention check, all participants were required to briefly turn on their cameras before the intervention began to confirm that they were physically present. Once attendance had been taken, they were asked to turn off their cameras and be attentive to the Examen-based process. Once the session was completed, participants were required to turn on their cameras once again to demonstrate their presence. To our knowledge, there was no interaction among the participants before or after the Examen-based intervention sessions. Measures were completed online by all participants at three time points: (1) upon signing up for the study (“pre-test”), (2) two weeks after beginning the study (“post-test”), and (3) four weeks after beginning the study (“follow-up”). All participants received credit toward completion of their introductory psychology course requirements. In addition, because participants in the examine-based intervention condition were asked to attend two weeks of evening Zoom sessions, they were awarded a $10 Amazon gift card for their participation.

### Measures

#### State Hope Scale (SHS)

The SHS (Snyder et al., 1996) is a state-like measure of hope. The SHS consists of 6 items, half assessing agency thinking and half assessing pathways thinking. Sample items include, “At the present time, I am energetically pursuing my goals” (agency) and “I can think of many ways to reach my current goals” (pathways). Respondents indicate the extent to which each statement applies to them “right now” on a scale ranging from 1 (definitely false) to 8 (definitely true). Items are summed to yield a total score, with higher scores indicating greater levels of hope. Research supports the reliability and validity of the SHS (Snyder et al., 1996). The SHS was administered at all three time points. In the present sample, the SHS had a Cronbach’s alphas ranged between 0.81 and 0.91, across the three time points.

#### Meaning in Life Questionnaire (MLQ)

The Meaning in Life Questionnaire (MLQ) is a 10-item self-report measure of two constructs: “the search for meaning” (sample item includes, “I am always looking to find my life’s purpose”) and “the presence of meaning” (sample items includes, “I have discovered a satisfying life purpose”). Respondents are instructed to “take a moment to think about what makes your life feel important to you.” No specific time frame for this evaluation is given in the instructions. The MLQ uses a seven-point Likert scale ranging from 1 (absolutely true) to 7 (absolutely untrue). Items are summed to yield a scores on each subscale, with higher scores indicating greater levels of the presence or search for meaning, respectively. The MLQ was administered at all three time points. In past research, the MLQ has shown internal reliability consistency, as well as temporal and factor structure stability (Rose et al., 2017; Steger et al., 2006). In the present sample, the MLQ Presence scale had Cronbach’s alphas range between 0.86 and 0.91, and the MLQ Search scale had Cronbach’s alphas range between 0.87 and 0.89, across the three time points.

#### Mindful Attention Awareness Scale (MAAS)

The Mindful Attention Awareness Scale (MASS) is a 15-item self-report measure of trait mindfulness (Brown & Ryan, 2003). According to the developers of the scale, mindfulness involves an open or receptive awareness and attention. Sample items include “I find it difficult to stay focused on what’s happening in the present” and “I break or spill things because of carelessness, not paying attention, or thinking of something else.” Participants respond on a five-point Likert scale ranging from 1 (almost always) to 6 (almost never) regarding how frequently or infrequently they currently experience mindfulness as described in each item. No specific time frame is given in the scale’s instructions for participants to make this evaluation beyond the word “currently.” Consistent with past studies (Brown & Ryan, 2003), items are averaged to yield a total score, with higher scores indicating greater levels of mindfulness. Past research shows that the MAAS has a one factor structure and has demonstrated reliability and validity (Park et al., 2013). The MAAS was administered at all three time points. In the present sample, Cronbach’s alphas ranged between 0.88 and 0.91.

#### Depression, Anxiety, and Stress Scale, 21-item Form (DASS-21)

The DASS-21 (Lovibond & Lovibond, 1995) is a 21-item measure with subscales for depression (DASS-D), anxiety (DASS-A), and stress (DASS-S) symptoms. Respondents indicate how much they experienced the symptom described by each item over the past week on a scale ranging from 0 (did not apply to me at all) to 3 (applied to me very much, or most of the time). Items are summed to yield scores for each subscale, with higher scores indicating greater symptoms of depression, anxiety, or stress, respectively. Sample items are “I couldn’t seem to experience any positive feeling at all” (depression), “I was worried about situations in which I might panic and make a fool of myself” (anxiety), and “I found it difficult to relax” (stress). Research supports the reliability and validity of the DASS-21 (Antony et al., 1998; Lovibond & Lovibond, 1995). The DASS-21 was administered at all three time points. In the present sample, Cronbach’s alphas ranged from 0.87 to 0.89 for depression, 0.79–0.83 for anxiety, and 0.77–0.82 for stress, across the three time points.

#### Santa Clara Brief Compassion Scale (SCBCS)

The Santa Clara Brief Compassion Scale (SCBCS; Hwang et al., 2008) consists of five self-descriptive items answered on a seven-point Likert scale ranging from 1 (not at all true of me) to 7 (very true of me). Participants are instructed to provide responses “as you feel right now.” A sample item includes, “I feel compassion for people, even though I do not know them.” Items are summed to yield a total score, with higher scores indicating greater levels of compassion. The SCBCS has demonstrated reliability and validity in past research (Hwang et al., 2008; Plante, 2022). In the present sample, Cronbach’s alphas ranged between 0.78 and 0.91.

#### Satisfaction with Life Scale (SWLS)

The SWLS (Diener et al., 1985) is a 5-item measure of general satisfaction with life. Items consist of statements about life (e.g., “In most ways my life is close to my ideal”), which respondents rate on a scale ranging from 1 (strongly disagree) to 7 (strongly agree). No particular time frame is given in the instructions for making these evaluations of life. Items are summed to yield a total score, with higher scores indicating greater levels of life satisfaction. Research supports the reliability and validity of the SWLS (Diener et al., 1985). The SWLS was administered at all three time points. In the present sample, Cronbach’s alphas ranged between 0.83 and 85.

#### Was-It-Worth-It Scale (WIWI)

The WIWI is a 5-item measure developed to assess the degree to which participants in research studies find the experience worthwhile (Sloan et al., 2011). It was originally used in cancer settings, including in the Mayo Clinic phase I research program, as a way to evaluate patient perceptions of the value of clinical trial participation (Thanarajasingam et al., 2022). However, it has since been used in psychosocial treatment applications (Corn et al., 2023). All items are verbatim from the original measure, except that the word “Examen” was substituted for “this cancer treatment.” Three items are answered yes, no, or undecided. These items were “Was it worthwhile for you to participate in the Examen?”, “If you had it to do over, would you participate in the Examen again?”, and “Would you recommend participating in the Examen to others?”. The remaining 2 items are answered on scales. These items were “Overall, how was your experience of participating in the Examen?” (with anchors of “1. Worse than I expected,” “2. Same as I expected,” and “3. Better than I expected”), and “Overall, do you believe your quality of life has increased by participating in the Examen?” (with anchors of “1. It got worse,” “2. It stayed the same,” and “3. It improved”). The WIWI was administered only to those who participated in the Examen-based intervention at follow-up (i.e., Time 3), given that it assesses reactions to the intervention. Because each WIWI item is typically analyzed separately, no overall Cronbach’s alpha can be computed.

### Data Analyses

All analyses were performed using the SPSS (version 29). Continuous variables were examined for skewness and kurtosis. Means, standard deviations, and counts/frequencies were used to characterize demographics of the sample as well as participants’ responses to the items on the WIWI measure. To test for the effects of the Examen-based intervention, we used a series of 2 (condition: intervention, wait-list control) × 3 (time: pre-test, post-test, follow-up) mixed ANOVAs. Because this was an exploratory study of the possible effects of the Examen-based intervention on a relatively large array of outcomes, no adjustment for multiple comparisons was used. Thus, for all analyses, the critical p-value for statistical significance was set to 0.05.

## Results

We hypothesized that participants in the Examen-based intervention condition would report increased levels of hope (SHS), meaning in life (both MLQ Presence of Meaning and MLQ Search for Meaning), compassion (SCBCS), mindfulness (MAAS), and life satisfaction (SWLS), as well as decreased levels of depression, anxiety, and stress (DASS-21), relative to those who did not receive the intervention. As mentioned, a series of 2 (condition: intervention, wait-list control) × 3 (time: pre-test, post-test, follow-up) mixed ANOVAs were used to test these hypotheses. Prior to analysis, all variables were examined for assumption of statistical normality (i.e., kurtosis < 4 and skewness < 2). All variables met these assumptions, with skewness ranging from − 0.789 to 0.931, and kurtosis ranging from − 1.083 to 0.961. In addition, participant attrition was examined. Though 115 individuals filled out pre-test measures, a total of 109 completed post-test measures, and 108 completed all measures at all three time points. This represents 93.9% participant retention by follow-up. Table 3 contains means and standard deviations for all study measures. For the DASS-21, in particular, researchers have established cutoff scores (Lovibond & Lovibond, 1995). At pre-test, 56.1%, 76.3%, and 55.3% of participants exceeded cutoff values for the “normal” range of scores on the depression, anxiety, and stress scales, respectively.

**Table 3:** Means and standard deviations of study measures

	Time 1		Time 2		Time 3	
	Examen *M* (SD)	Control *M* (SD)	Examen *M* (SD)	Control *M* (SD)	Examen *M* (SD)	Control *M* (SD)
DASS depression	12.74 (9.16)	11.24 (9.25)	13.04 (9.41)	12.52 (9.77)	12.33 (9.79)	12.06 (9.32)
DASS anxiety	14.37 (8.99)	14.18 (9.60)	13.74 (10.27)	13.71 (8.75)	13.74 (9.22)	13.27 (9.30)
DASS stress	17.11 (7.69)	16.26 (8.65)	16.89 (8.83)	17.16 (8.81)	15.82 (8.34)	16.95 (8.61)
SHS total hope	35.06 (5.84)	35.00 (5.86)	35.28 (6.76)	33.08 (6.43)	35.41 (6.62)	32.33 (7.96)
MLQ presence	21.83 (5.83)	22.22 (5.63)	23.00 (5.79)	21.42 (6.72)	22.87 (6.01)	21.16 (6.77)
MLQ search	24.38 (6.43)	23.82 (5.15)	25.85 (5.61)	24.27 (5.86)	26.40 (5.26)	23.89 (6.04)
SWLS	22.60 (6.29)	23.80 (5.46)	23.28 (6.57)	22.91 (5.79)	23.69 (6.20)	22.91 (5.96)
SCBCS	27.56 (4.77)	25.98 (5.43)	27.83 (4.87)	25.44 (5.03)	26.80 (4.99)	25.40 (5.49)
MAAS (reversed)	3.68 (0.74)	3.66 (0.80)	3.65 (0.56)	3.66 (0.87)	3.63 (0.67)	3.70 (0.94)

As mentioned, we performed a series of 2 (condition: Examen-based practice, wait-list control) × 3 (time: pre-test, post-test, follow-up) mixed ANOVAs to test for the effects of our intervention over time (see Table 4). The interaction reached significance in the analyses predicting MLQ Presence of Meaning scores (*F* (2, 212) = 3.57, *p* = 0.030, partial eta^2^ = 0.033), SHS scores (*F* (2, 214) = 4.77, *p* = 0.009, partial eta^2^ = 0.043), and SWLS scores (*F* (2, 212) = 3.74, *p* = 0.025, partial eta^2^ = 0.034). The interaction approached significance with regard to predicting MLQ Search for Meaning scores (*F* (2, 212) = 2.57, *p* = 0.079, partial eta^2^ = 0.024). However, the interaction was not significant in the analyses predicting DASS Depression scores (*F* (2, 214) = 0.334, *p* = 0.716), DASS Anxiety scores (*F* (2, 214) = 0.040, *p* = 0.961), DASS Stress scores (*F* (2, 214) = 0.903, *p* = 0.407), SCBCS scores (*F* (2, 214) = 1.17, *p* = 0.313), or MAAS scores (*F* (2, 212) = 0.384, *p* = 0.682).

**Table 4:** Results of 2 (condition: Examen-based intervention, control) × 2 (time: pre-test, post-test, follow-up) mixed model ANOVAs (*N* = 108)

Outcome	*F*	*DV*_1_	*DV*_2_	*p*	$\eta$^2^
MLQ presence	3.57	2	212	.030	.033
MLQ search	2.57	2	212	.079	.024
SHS	4.77	2	212	.009	.043
SWLS	3.74	2	212	.025	.034
DASS depression	0.33	2	214	.716	.003
DASS anxiety	0.04	2	214	.961	.001
DASS stress	0.90	2	214	.407	.008
SCBCS	1.17	2	214	.313	.011
MAAS	0.38	2	212	.682	.004

The parameters reported for each outcome measure represent the test of the condition x time interaction

Marginal means for all study measures across condition and time can be found in Table 3. We performed post hoc analyses to probe these means. For MLQ Presence of Meaning, the interaction appeared to be driven by the fact that scores increased in the Examen-based intervention condition (*p* = 0.06) from pre-test to post-test, and then were maintained from post-test to follow-up (*p* = 0.82). This was similar for MLQ Search for Meaning, with scores increasing from pre-test to post-test in the intervention condition (*p* = 0.03), and remaining stable from post-test to follow-up (*p* = 0.33). Scores did not change over time in the control condition for either of these sets of analyses. The pattern for SWLS scores was somewhat different, with no significant change in scores from pre-test to post-test (*p* = 0.24), but an overall increase from pre-test to follow-up (*p* = 0.06) in the intervention condition. Again, no significant changes in scores over time were noted in the control condition. Finally, for the SHS, the interaction appeared to be drive by a decrease in scores from pre-test to post-test in the control group (*p*. = 0.01), with this decrease maintained from post-test to follow-up (*p* = 0.24). In this analysis, scores remained stable and relatively high in the intervention group.

We also were interested in how the 57 participants who took part in the Examen-based intervention would evaluate their experience on the WIWI scale. Responses can be found in Table 5. Nearly all participants answered “yes” to the items “Was it worthwhile to participate in the Examen?”, “If you had to do it over, would you participate in the Examen again?”, and “Would you recommend participating in the Examen to others?” (see Table 1). Recall that two additional WIWI items were reported on 3-point scales, where higher scores indicate higher/better ratings. These items were “Overall, how was your experience of participating in the Examen?” and, “Overall, do you believe your quality of life has increased by participating in the Examen?”, which had means of 2.66 (SD = 0.48) and 2.47 (SD = 0.50), respectively.

**Table 5:** Ratings on “Was-it-Worth-it” (WIWI) Items at Follow-up (*N* = 57)

Item	Yes	No	Undecided	Missing Response	*χ*^*2*^	*p*
“Was it worthwhile for you to participate in the Examen?”	43 (75.4%)	2 (3.5%)	8 (14.0%)	4 (7.0%)	78.65	< .001
“If you had it to do over, would you participate in the Examen again?”	44 (77.2%)	3 (5.3%)	6 (10.5%)	4 (7.0%)	83.14	< .001
“Would you recommend participating in the Examen to others?”	44 (77.2%)	1 (1.8%)	8 (14.0%)	4 (7.0%)	84.54	< .001

## Discussion

The purpose of the present study was to explore the possible effects of an Examen-based practice on a relatively wide array of outcomes. We found that engaging in the Examen-based practice on a daily basis for two weeks appeared to improve participants’ levels of life satisfaction and presence of life meaning relative to a wait-list control group. There was also an effect approaching statistical significance on search for life meaning. The effect sizes for all of these analyses were in the small-to-medium range, with partial eta-squares from 0.024 to 0.034. Thus, an Examen-based practice appears to encourage individuals to evaluate their lives more positively in a global sense, perhaps “counting their blessings” by focusing on satisfying or meaningful aspects of their daily existence.

In addition, the largest effect size was found for hope (partial eta-squared of 0.043). In this case, however, the Examen-based practice appears to have helped maintain individuals’ perceptions of their abilities to strive for goals (i.e., hope), leading to a more stable positive view of the future over time. This contrasts with participants in the control condition, whose hope decreased during the study. In university students, hope has been shown to be a robust predictor of better academic performance, higher graduate rates, and greater probability general goal accomplishment (Feldman & Kubota, 2015; Feldman et al., 2009; Snyder et al., 2002). Although interventions exist to nurture hope (e.g., Cheavens et al., 2006; Feldman & Dreher, 2012), the Examen-based practice’s very brief, daily nature may offer a more flexible approach than longer sessions.

Counter to expectations, we did not find effects of the intervention on mindfulness. It may be that the Examen-based practice’s emphasis on examining the day is too “past-focused” to encourage an increase in mindfulness, which tends to be explicitly present-moment-focused. The lack of effect may also be due to the particular measure of mindfulness utilized in the present study—the Mindful Attention Awareness Scale (MAAS). The MAAS is often described as a “dispositional” or “trait” measure of hope (Brown & Ryan, 2003), and thus may not be amenable to short-term intervention. Future studies employing longer-term versions of the Examen-based practice (perhaps for several months) or differing measures of mindfulness may be helpful in clarifying these issues.

We also did not find effects for stress, anxiety, or depression. One explanation for this lack of effects may be that participants were not selected for heightened distress, thus not allowing sufficient room for improved scores. However, it should be noted that, according to established norms (Lovibond & Lovibond, 1995), pre-test means on the DASS-21 were in the mild to moderate ranges (rather than the “normal” range) for the depression, anxiety, and stress subscales, ruling out absolute floor effects. It is curious, however, that these measures are also relatively affective in nature (which was likewise true of our compassion measure), whereas the measures on which the Examen-based practice had effects (i.e., satisfaction with life, meaning in life, hope) are all more cognitive. Perhaps the instructions for the intervention utilized in the present study encouraged participants to dwell more in the “head” than the “heart.” Of note, dozens of versions of the Examen have been developed (Thibodeaux, 2014). Though all of these follow the same basic steps, their details differ. The particular “secularized” instructions employed in the present study attempted to mitigate some of the traditional religious or spiritual language present in some versions of the Examen. It may be that, in this process of secularization, some of the potential affective impact of the practice was reduced.

## Limitations

As with all studies, the present research has limitations. These include the use of college students as research participants, the reliance on self-report measures, and the use of a wait-list control condition. As such, the present study cannot determine how participating in an Examen-based practice might compare to other daily practices such as mindful meditation, yoga, or prayer. Furthermore, it is unclear if a 15-min per day, two-week intervention was adequate or if a more extensive intervention, including more weeks of treatment, may result in stronger outcomes.

Additional limitations include the high proportion of female versus male participants, the lack of follow-up information beyond our two-week assessment, relatively small effect sizes, the exploratory nature of the analyses, and the absence of additional information about other potentially relevant outcomes. In addition, the Examen-based intervention participants received a $10 gift card while the control group did not. Finally, while attendance was noted and participants were instructed to turn on their cameras at the start and end of each session, it is unclear how attentive they were to the Examen-based practice itself.

## Future Research

In the future, researchers should consider comparing the Examen-based practice to other potentially helpful interventions, possibly including mindfulness, yoga, relaxation training, prayer, or cognitive-behavioral therapy. There are a variety of spiritual, religious, and contemplative practices that have been successfully research and integrated into clinical practice (Kabat-Zinn, 2003; Rosmarian, 2018; Segal et al., 2018). Perhaps the Examen and Examen-based practices could be further investigated and potentially compared to these more researched and established interventions. Additionally, future research can probe the generalizability of the present findings by incorporating a greater diversity of populations, including samples of individuals drawn from cultures across the globe, as well as older adults, children, and possibly clinical populations (e.g., those experiencing depression, anxiety, personality disorders, or substance use disorders). It may also be useful to consider which scales best capture the effects of the Examen-based intervention. In the present study, we found effects on measures that are cognitive in nature and concern evaluations of one’s goals and/or projects in life (i.e., hope, life satisfaction, and meaning in life). In the future, researchers may wish to consider using additional measures that reflect this general stance, including scales of self-efficacy (Schwarzer & Jerusalem, 1995), self-esteem (Rosenberg, 1979), optimism (Scheier et al., 2001), or possible selves (Markus & Nurius, 1986). Finally, researchers could explore whether the Examen-based practice is more useful when administered in group or individual settings.

## Conclusions

The Examen has been a staple of Jesuit spirituality for more than 500 years. Yet only recently has it been subjected to empirical investigation. Much research is needed to determine if the Examen and Examen-based practices may be helpful in improving psychological functioning among those who choose to practice the technique. If so, it potentially adds to our toolkit of spiritual and secular techniques for increasing a sense of satisfaction, meaning, and hope amidst an increasingly challenging world.

## Appendix: Secular Examen-Based Practice

### Beginning (1 minute)

I invite you to take three slow, deep breaths at your own pace—just slowly become aware of your connection to yourself, this room, this group of people, this moment, and the world around you. We move to step one of our Examen.

### Step 1: Gratitude (2 minutes)

Even if today has felt hard, take some time to consider what good things (possibly “what has been life-giving today”) you have experienced today. Consider the really big ones (your life, safety, love) to the really small ones (a good night's sleep, an affirming conversation, a task completed, a compliment paid to you). For each gift that comes to mind, spend a moment giving thanks. We now move to step two of our Examen.

### Step 2: Asking for some help/Approaching the day with humility (1 minute)

You are invited now to ask for the clarity to see the truths of the day that you just experienced: the experiences that were life-giving, the moments that were life-draining; the joys and sorrows; the healthy and unhealthy. Just ask for some help for the rest of this Examen to see your day for what it truly is. And now we move to step three of our Examen.

### Step 3: Review and relive your day (4 minutes).

I invite you now to use your imagination to review and relive your day. I will walk you through the day, hour by hour. When significant moments arise, linger there for a while. If you notice yourself getting stuck on one aspect of your day, acknowledge its significance and gently let go as you continue to review your day. Start with the moment you woke up. What were those first things you did after waking up? What was that time like for you? And what came next? Try to stay grounded in your five senses as you relive the day. What about midday or lunchtime? What about the hours leading up to this Examen? What significant moments were there for you? Let’s move to step four.

### Step 4: Face the challenges of the day (3 minutes)

What were the difficult moments of the day? Moments when you had unhelpful thought patterns, when you said something hurtful, when you did something hurtful, or when you were hurt? Were there any missed opportunities, such as when you could have acted with more compassion? With this moment or moments in your mind and heart, consider if there is an invitation for healing, forgiveness, or reconciliation. Allow peace and love to wash over you. And we move on now to the last step of our Examen.

### Step 5: Look toward what’s next (2 minutes)

With what you have learned during this Examen about yourself and your life, is there anything you feel invited to do tomorrow? Perhaps more importantly, consider what sort of person you feel called to be tomorrow. Take a moment to resolve to be that person. You might even make a commitment to that effect. Or would you like to set some goals for tomorrow? And ask for the help you need to fulfill this commitment.

## Conclusion (1 minute)

And to conclude this Examen, I invite you to consider if there are any final things you want to express. Is there an intention you want to set? Take a few deep breaths.
